# An early evaluation of MedSigLIP in thyroid cytology: a comparative frozen-encoder benchmark against ImageNet-pretrained encoders

**DOI:** 10.3389/fendo.2026.1800630

**Published:** 2026-04-10

**Authors:** Mehmet Poyrazer, Rıdvan Erten

**Affiliations:** 1 Division of Endocrinology and Metabolism, University of Health Sciences Ankara Training and Research Hospital, Ankara, Türkiye; 2 Division of Geriatrics, Department of Internal Medicine, Faculty of Medicine, Kocaeli University, Kocaeli, Türkiye

**Keywords:** Bethesda system, deep learning, transfer learning, foundation models, MedSigLIP, model calibration, thyroid cytology

## Abstract

**Background:**

Fine-needle aspiration biopsy (FNAB) cytology is central to thyroid nodule evaluation, yet reliable differentiation across Bethesda categories remains challenging, particularly for the indeterminate Bethesda V (Suspicious for Malignancy) class. While transfer learning with ImageNet-pretrained models is a standard approach, it remains unclear whether emerging domain-specific medical foundation models offer superior performance compared to general purpose baselines in this specialized domain.

**Methods:**

We benchmarked four frozen visual encoders—ResNet50, EfficientNet-B0, ViT-Base (ImageNet pretrained), and MedSigLIP (medical image–text pretrained)—on the ThyroidEffi 1.0 dataset (N = 1,804), comprising Bethesda II (Benign), Bethesda V (Suspicious), and Bethesda VI (Malignant) cases. A unified evaluation protocol was employed using five-fold stratified cross-validation with a lightweight multilayer perceptron head. Performance was assessed using macro-F1, balanced accuracy, Expected Calibration Error (ECE), and McNemar’s test for statistical significance.

**Results:**

EfficientNet achieved the highest macro-F1 (0.845 ± 0.021), followed closely by MedSigLIP (0.836 ± 0.019), ResNet50 (0.829 ± 0.015), and ViT (0.817 ± 0.020). Pairwise statistical testing revealed that while EfficientNet significantly outperformed ViT (p < 0.05), the difference between EfficientNet and MedSigLIP was not statistically significant after multiple comparison correction. Notably, MedSigLIP demonstrated superior reliability attributes, achieving the highest recall for the challenging Suspicious class (0.808) and the best model calibration score (ECE = 0.025) compared to the general-purpose encoders (ECE: 0.044–0.082).

**Conclusions:**

While domain-specific medical pretraining (MedSigLIP) did not yield a statistically significant advantage in aggregate classification accuracy compared to the best ImageNet-based model (EfficientNet), it provided superior calibration and sensitivity for borderline cases. These findings suggest that in thyroid cytology clinical workflow support, encoder selection should be guided by a joint view of discrimination and safety—particularly calibration and Bethesda V sensitivity—rather than aggregate accuracy alone, enabling threshold-based triage and selective expert review. In particular, well-calibrated models such as MedSigLIP suggest a potential benefit in reducing overconfident misclassification in borderline Bethesda V cases, pending prospective validation in real-world triage workflows.

## 1 Introduction

Thyroid nodules are among the most common endocrine conditions, with ultrasound detected nodules reported in up to half of the general population (1). While the majority of these nodules are benign, a clinically meaningful minority harbor malignancy, making accurate risk stratification essential for guiding management and avoiding unnecessary surgery (2). Fine-needle aspiration biopsy (FNAB) cytology remains the cornerstone for preoperative evaluation, providing a minimally invasive pathway for categorizing malignancy risk and informing treatment decisions (3). Trends indicate that while thyroid cancer incidence has stabilized, the clinical burden of indeterminate nodules remains high (4).

To standardize reporting and downstream clinical decision making, the Bethesda System for Reporting Thyroid Cytopathology (TBSRTC) classifies FNAB cytology into six categories ranging from benign (Bethesda II) to malignant (Bethesda VI) (5). Although this framework has significantly improved communication between cytopathologists and clinicians, diagnostic uncertainty persists, particularly in borderline or “gray-zone” cases (6). Indeterminate categories (Bethesda III–IV) are frequent and often necessitate diagnostic lobectomies or molecular testing to refine risk estimates (7, 8). Bethesda V (Suspicious for Malignancy) presents a specific challenge: cytology exhibits features highly suggestive of malignancy but lacks definitive criteria, resulting in substantial uncertainty despite a relatively high risk of malignancy (5).

A major contributor to this uncertainty is interobserver variability, which is most pronounced in intermediate categories where microscopic criteria are inherently subjective. Multi-institutional evidence suggests that variability in practice patterns and diagnostic thresholds can meaningfully shift downstream risk profiles, emphasizing the need for more objective and reproducible decision support (9). Recent efforts to improve reproducibility through refined subclassification schemes further underline that standardization alone does not fully resolve diagnostic ambiguity in these borderline categories (8, 10).

In parallel, deep learning has emerged as a promising approach for assisting cytology and broader thyroid imaging workflows, including triage support and Bethesda category prediction (11, 12). Yet, clinical translation remains limited by real-world barriers such as staining variability, scanner heterogeneity, and domain shift across institutions—factors that can substantially degrade generalization (11). Moreover, even when performance is high on average, suspicious or ambiguous cytology categories remain difficult, frequently yielding lower class-wise sensitivity than benign or malignant cases in prior thyroid AI literature (13, 14).

​Transfer learning has become a default strategy in medical imaging because it enables effective feature extraction even when task specific labeled datasets are limited (15). In practice, this typically involves using general purpose encoders pretrained on large natural image corpora such as ImageNet-1K or ImageNet-21K (16, 17). However, the transferability of natural image features to histopathology—where textures and cellular patterns differ fundamentally from macroscopic objects—remains a subject of debate (18). More recently, domain-specific “medical foundation” encoders have been introduced to bridge this gap. MedSigLIP, developed within Google’s Health AI Developer Foundations, is a vision-language encoder derived from SigLIP and pretrained on tens of millions of medical image–text pairs (19–21). Despite the theoretical advantages of medical pretraining, direct head-to-head comparisons between such domain-specific encoders and standard ImageNet-pretrained models remain limited for thyroid cytology (22).

To our knowledge, thyroid FNAB cytology has not been systematically benchmarked under a unified frozen-encoder protocol that (i) compares a domain-specific medical vision–language encoder with standard ImageNet-pretrained backbones, (ii) uses identical patient level folds for paired statistical testing, and (iii) reports calibration behavior alongside discrimination. Therefore, we designed a controlled representation transfer benchmark with a lightweight MLP head and evaluated not only macro-F1 but also confidence reliability (ECE) and borderline category performance (Bethesda V).

​From a methodological standpoint, evaluating “frozen” encoders—keeping the pretrained feature extractor fixed and training only a lightweight classifier head—offers a rigorous way to benchmark the quality of transferable representations without the confounding effects of full fine-tuning (18, 23). Furthermore, beyond classification accuracy, the reliability of confidence estimates is critical for clinical decision support. Poorly calibrated models can assign high confidence to incorrect predictions, which may be particularly hazardous in borderline categories such as Bethesda V (24). Consequently, calibration and uncertainty quantification have become increasingly prioritized in medical AI, yet remain underreported in thyroid cytology studies (25, 26).

​Against this backdrop, we benchmark four representative frozen visual encoders—ResNet50 (27), EfficientNet-B0 (28), ViT-Base (29), and MedSigLIP (19)—on the ThyroidEffi 1.0 dataset (30), a multi-class FNAB cytology benchmark comprising Bethesda II (Benign), Bethesda V (Suspicious), and Bethesda VI (Malignant) images. Our primary objective is to compare macro-F1 performance under a unified cross-validation protocol. Secondary objectives include a detailed analysis of error patterns in suspicious cases, assessment of model calibration using Expected Calibration Error (ECE), and evaluation of robustness under simulated image perturbations. Rather than proposing a novel architecture, this study contributes a methodologically rigorous benchmark protocol, with emphasis on patient-level stratification, paired statistical testing, and calibration analysis as evaluation criteria.

## 2 Materials and methods

### 2.1 Dataset description

This study utilized the ThyroidEffi 1.0 dataset (30), which was obtained from the original authors upon reasonable request. The dataset consists of Diff-Quik–stained thyroid FNAB cytology images acquired at 40× magnification using an Olympus BX43 microscope. The dataset comprises 1,804 images from histopathologically confirmed cases, grouped into three Bethesda categories: Bethesda II (Benign, n = 482, 26.7%), Bethesda V (Suspicious for Malignancy, n = 541, 30.0%), and Bethesda VI (Malignant, n = 781, 43.3%). Each image corresponds to a unique patient case, ensuring that no patient level overlap existed across cross-validation folds. The dataset inherently contains one image per patient; no multiple image per patient exclusion was required. All encoders were evaluated on the same patient-level folds and identical train/validation/test partitions to enable strictly paired, fair comparisons across models.

### 2.2 Frozen visual encoders and embedding extraction

We benchmarked four pretrained visual encoders representing widely used general purpose and domain-specific paradigms: ResNet50 (27), EfficientNet-B0 (28), ViT-Base (29), and MedSigLIP (medical image–text pretraining) (19). Encoders were utilized in a frozen setting to isolate representation quality and reduce computational confounding from full fine-tuning.

All images were loaded from class-labeled directories and mapped to integer labels (Benign: 0, Suspicious: 1, Malignant: 2). For ImageNet-pretrained models (ResNet50, EfficientNet, ViT), images were resized to 224×224 pixels and normalized using standard ImageNet mean and standard deviation. For MedSigLIP, images were resized to 448×448 pixels and processed using the model’s native image processor and normalization parameters to align with its pretraining protocol (19–21). Although this resolution difference follows each model’s native preprocessing protocol, it may introduce a subtle benchmarking confound; comparisons should therefore be interpreted with this caveat in mind. MedSigLIP was evaluated in pure vision mode; only the vision encoder was used for embedding extraction, without any text input or multimodal fusion. Embeddings were extracted for each image and cached to disk to ensure identical input data across all classifier training runs.

### 2.3 Classification head and training protocol

**Figure 1:**
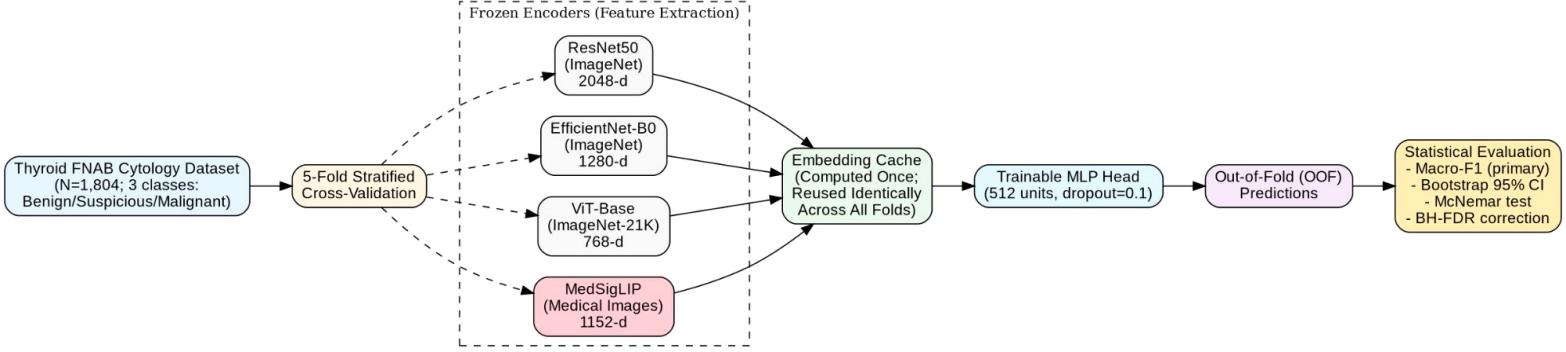
Overview of the study workflow. Thyroid FNAB cytology images from the ThyroidEffi 1.0 dataset (Bethesda II/V/VI; Benign/Suspicious/Malignant) were preprocessed and passed through four frozen pretrained encoders (ResNet50, EfficientNet-B0, ViT-Base, and MedSigLIP) to extract fixed-dimensional embeddings. A lightweight MLP classifier head (encoder_dim → 512 → 3; dropout = 0.1) was trained on cached embeddings using 5-fold stratified cross-validation, with an internal validation split for early stopping. Performance was summarized using macro-F1 (primary) and balanced accuracy, with additional analyses including paired model comparisons (paired bootstrap and McNemar tests with BH–FDR adjustment), class-wise error inspection focusing on Bethesda V, robustness to common image perturbations, and calibration assessment. FNAB, fine-needle aspiration biopsy; MLP, multilayer perceptron; OOF, out-of-fold; BH–FDR, Benjamini–Hochberg false discovery rate.

The overall experimental pipeline, illustrating the frozen encoder extraction and MLP classification framework, is presented in **Figure 1**. Frozen encoder embeddings were passed to a lightweight classification head implemented as a two layer multilayer perceptron (MLP): [encoder_dim] → 512 → 3, with ReLU activation. The encoder output dimensionality depended on the backbone architecture (2048 for ResNet50, 1280 for EfficientNet-B0, 768 for ViT-Base, and 1152 for MedSigLIP). Dropout (p = 0.1) was applied after the first MLP layer to mitigate overfitting.

Model evaluation employed a five-fold stratified cross-validation scheme to preserve class proportions across folds. Within each training fold, 15% of the training data was reserved as a validation split for model selection. The classifier head was optimized using the AdamW optimizer with an initial learning rate of 1×10^-^³, weight decay of 0.01, and a batch size of 64. Training ran for up to 30 epochs with early stopping (patience = 5 epochs) monitoring validation macro-F1. A cosine annealing learning rate scheduler was applied across epochs.

To address class imbalance, a weighted cross entropy loss was employed. Class weights were computed within each fold from the training labels using the “balanced” inverse frequency heuristic. Since encoders were frozen and trained on cached embeddings, image space augmentation was not applied during head training; however, robustness to realistic perturbations was evaluated separately (see Section 2.6). All hyperparameters (learning rate, dropout, weight decay) were fixed *a priori* and applied identically across all encoders; no encoder-specific tuning was performed, ensuring strict fairness in the benchmark.

### 2.4 Evaluation metrics

The primary performance metric was the macro averaged F1-score (macro-F1), which provides a balanced assessment of performance across classes despite dataset imbalance. Secondary metrics included balanced accuracy, class-wise precision, recall, F1-scores, and confusion matrices. Model level comparisons focused on out-of-fold (OOF) predictions aggregated across all cross-validation folds.

### 2.5 Statistical analysis

To quantify uncertainty in macro-F1, we computed bootstrap 95% confidence intervals (CIs) using 1000 resamples of the OOF predictions (31). For pairwise encoder comparisons, we reported (i) paired bootstrap differences in macro-F1 and (ii) McNemar’s test for paired nominal data (32). To control for multiplicity across the six possible encoder pairs, the Benjamini–Hochberg (BH) false discovery rate correction was applied to McNemar p values (α = 0.05) (33). All analyses were implemented using Python 3.10 and PyTorch 2.1 frameworks. Statistical tests were performed using the scipy.stats and statsmodels libraries. Paired bootstrap provides continuous effect size estimates with confidence intervals, while McNemar’s test evaluates discrete outcome agreement; in cases of discrepancy, BH-adjusted McNemar p-values were treated as the primary statistical result. Formal power analysis was not conducted *a priori*; however, given the observed Δ macro-F1 = 0.0084 between EfficientNet and MedSigLIP, the sample size (N = 1,804) provides adequate power to detect moderate-to-large differences, while small differences of this magnitude should be interpreted with caution. Using a paired bootstrap framework with N = 1,804, the bootstrap standard error of Δ macro-F1 between EfficientNet and MedSigLIP was 0.0086, yielding a minimum detectable difference of approximately 0.024 at 80% power and α = 0.05 (two-sided). The observed gap (Δ = 0.0084) falls well below this threshold, further supporting its interpretation as a clinically negligible difference. Paired bootstrap Δ macro-F1 with 95% confidence intervals (**Table 1**) are reported as the primary effect size measure for all pairwise comparisons.

**Table 1:** Pairwise encoder comparisons using out-of-fold (OOF) predictions.

Comparison	Δ Macro-F1	95% CI	Bootstrap p	McNemar p	McNemar p (BH-adj)
ResNet50 vs EfficientNet	-0.0163	[-0.0324, -0.0011]	0.04	0.1006	0.2012
ResNet50 vs ViT	0.0119	[-0.0058, +0.0296]	0.19	0.165	0.2476
ResNet50 vs MedSigLIP	-0.0076	[-0.0255, +0.0100]	0.414	0.6758	0.6758
EfficientNet vs ViT	0.0278	[+0.0107, +0.0458]	0.002	0.0016	0.0099
EfficientNet vs MedSigLIP	0.0084	[-0.0090, +0.0257]	0.322	0.2615	0.3138
ViT vs MedSigLIP	-0.0193	[-0.0391, -0.0002]	0.048	0.073	0.2012

Δ Macro-F1 = Macro-F1(first model in “Comparison”) − Macro-F1(second model). 95% CI denotes the paired bootstrap 95% confidence interval for Δ Macro-F1 computed from out-of-fold predictions (1,000 resamples). Bootstrap p-values are from paired bootstrap resampling. McNemar p-values are from exact binomial McNemar tests on paired correctness. BH-adj denotes Benjamini–Hochberg FDR-adjusted p-values across the six pairwise comparisons. CI, confidence interval; OOF, out-of-fold; BH, Benjamini–Hochberg; FDR, false discovery rate.

### 2.6 Calibration and robustness analyses

Calibration was assessed using the Expected Calibration Error (ECE) computed with a fixed-bin approach (10 bins) from predicted confidence scores (25). Reliability diagrams were generated to visualize calibration behavior. Robustness was evaluated under common real-world image perturbations (e.g., brightness shifts, Gaussian blur, JPEG compression) aligned with established “common corruption” robustness evaluation practices (34) using a single train/validation/test split to reflect deployment like conditions; perturbation specific macro-F1 scores were reported.

To assess robustness to binning strategy, Adaptive Calibration Error (ACE) was additionally computed using equal-mass bins. Multiclass Brier Score was calculated per class and averaged across classes. Class-wise ECE and ACE were separately reported for the Bethesda V (Suspicious) category.

## 3 Results

### 3.1 Overall performance comparison

The ThyroidEffi 1.0 dataset comprised 1,804 FNAB cytology images across three Bethesda categories: Bethesda II (Benign, n = 482, 26.7%), Bethesda V (Suspicious, n = 541, 30.0%), and Bethesda VI (Malignant, n = 781, 43.3%) (**Table 2**).

**Table 2:** Dataset summary and class distribution used for the 3-class benchmark.

Class	Bethesda Category	N	Percentage (%)	Train per fold (≈)	Test per fold (≈)
Benign	II	482	26.7	385	96
Suspicious	V	541	30.0	432	108
Malignant	VI	781	43.3	624	156
Total	–	1804	100.0	1443	360

Classes correspond to Bethesda II (Benign), Bethesda V (Suspicious), and Bethesda VI (Malignant). Counts and percentages are computed on the full dataset (N = 1804). Perfold sizes are approximate for 5-fold stratified cross-validation.

Across five-fold stratified cross-validation, EfficientNet achieved the highest overall macro-F1 (0.845 ± 0.021; 95% CI [0.829, 0.861]), followed closely by MedSigLIP (0.836 ± 0.019; 95% CI [0.821, 0.852]), ResNet50 (0.829 ± 0.015; 95% CI [0.812, 0.846]), and ViT (0.817 ± 0.020; 95% CI [0.801, 0.834]) (**Table 3**). A similar ranking was observed for balanced accuracy, with EfficientNet yielding the highest score (0.848 ± 0.021).

**Table 3:** Main out-of-fold (OOF) performance across 5-fold stratified cross-validation (mean ± SD across folds).

Encoder	Macro-F1	95% CI	Balanced Acc	Benign F1	Suspicious F1	Malignant F1
ResNet50	0.829 ± 0.015	[0.812, 0.846]	0.830 ± 0.008	0.928 ± 0.014	0.740 ± 0.023	0.819 ± 0.022
EfficientNet	0.845 ± 0.021	[0.829, 0.861]	0.848 ± 0.021	0.941 ± 0.007	0.764 ± 0.036	0.831 ± 0.025
ViT	0.817 ± 0.020	[0.801, 0.834]	0.823 ± 0.016	0.921 ± 0.024	0.732 ± 0.021	0.799 ± 0.034
MedSigLIP	0.836 ± 0.019	[0.821, 0.852]	0.840 ± 0.015	0.942 ± 0.012	0.750 ± 0.020	0.816 ± 0.041

95% CI denotes the bootstrap 95% confidence interval for Macro-F1 computed from out-of-fold (OOF) predictions (1,000 resamples). Balanced accuracy is defined as the mean recall across classes. Acc, accuracy; CI, confidence interval; OOF, out-of-fold.

**Figure 2:**
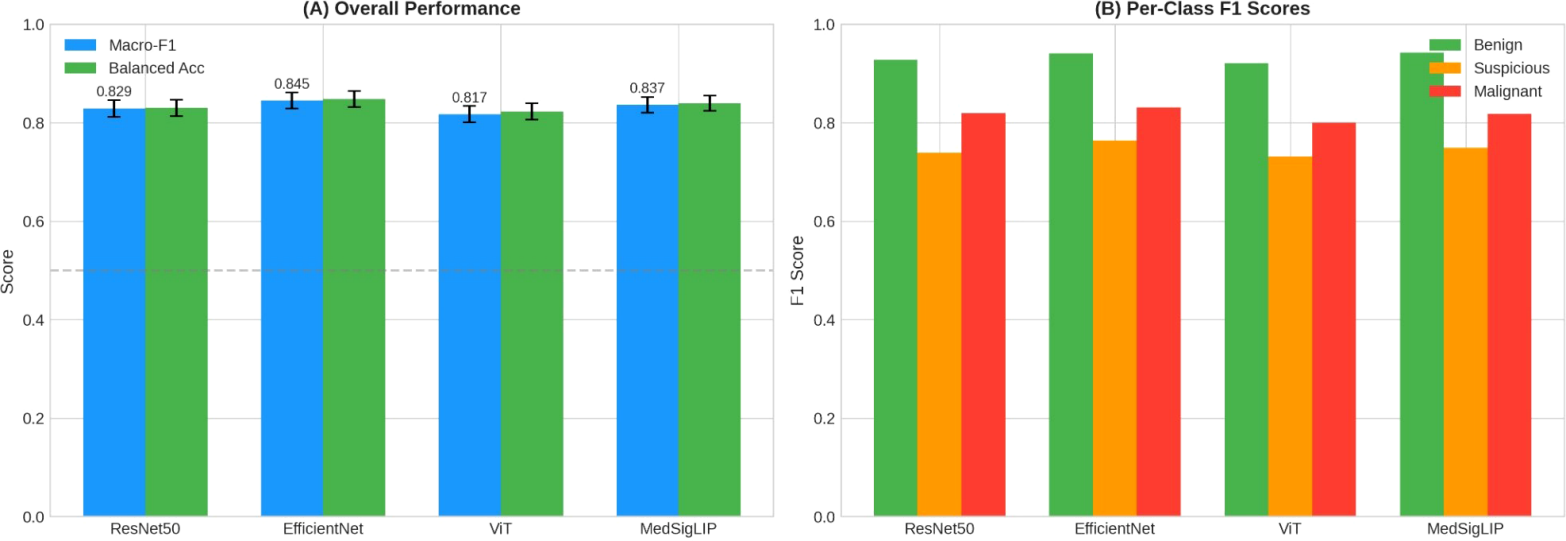
Overall and per-class performance across frozen encoders under 5-fold cross-validation. **(A)** Overall performance summarized by macro-F1 (primary) and balanced accuracy for each encoder under 5-fold stratified cross-validation. Bar heights represent the mean across folds and error bars indicate variability across folds (mean ± SD; see **Table 3** for numeric values). **(B)** Per-class F1 scores for Benign (Bethesda II), Suspicious (Bethesda V), and Malignant (Bethesda VI), averaged across folds. Overall, EfficientNet showed the highest macro-F1, followed closely by MedSigLIP and ResNet50, whereas ViT yielded the lowest macro-F1. SD, standard deviation.

Class-wise performance showed consistently high Benign F1 across encoders (0.921–0.942), while the diagnostically critical Suspicious class remained the most difficult (F1: 0.732–0.764). Malignant class performance ranged from 0.799 to 0.831 across encoders (**Table 3**). A summary comparison of macro-F1 across models is shown in **Figure 2**.

### 3.2 Statistical significance testing

Pairwise comparisons of encoder performance are summarized in **Table 1**. EfficientNet demonstrated a statistically significant advantage over ViT, with a paired bootstrap macro-F1 difference of +0.0278 (95% CI [+0.0107, +0.0458], bootstrap p = 0.002). This difference remained significant under paired-outcome testing (McNemar p = 0.0016; BH-adjusted p = 0.0099).

In contrast, the difference between EfficientNet and MedSigLIP was small and not statistically significant (Δ macro-F1 = +0.0084; 95% CI [−0.0090, +0.0257]; McNemar BH-adjusted p = 0.3138). Likewise, comparisons of ResNet50 versus ViT and ResNet50 versus MedSigLIP did not yield statistically significant differences after multiple comparison correction. A small bootstrap advantage was observed for EfficientNet versus ResNet50 (Δ macro-F1 = +0.0163; 95% CI [+0.0011, +0.0324]), although this was not supported by McNemar testing after correction (BH-adjusted p = 0.2012), suggesting that the observed performance gap should be interpreted cautiously.

### 3.3 Confusion matrices and error patterns

**Figure 3:**
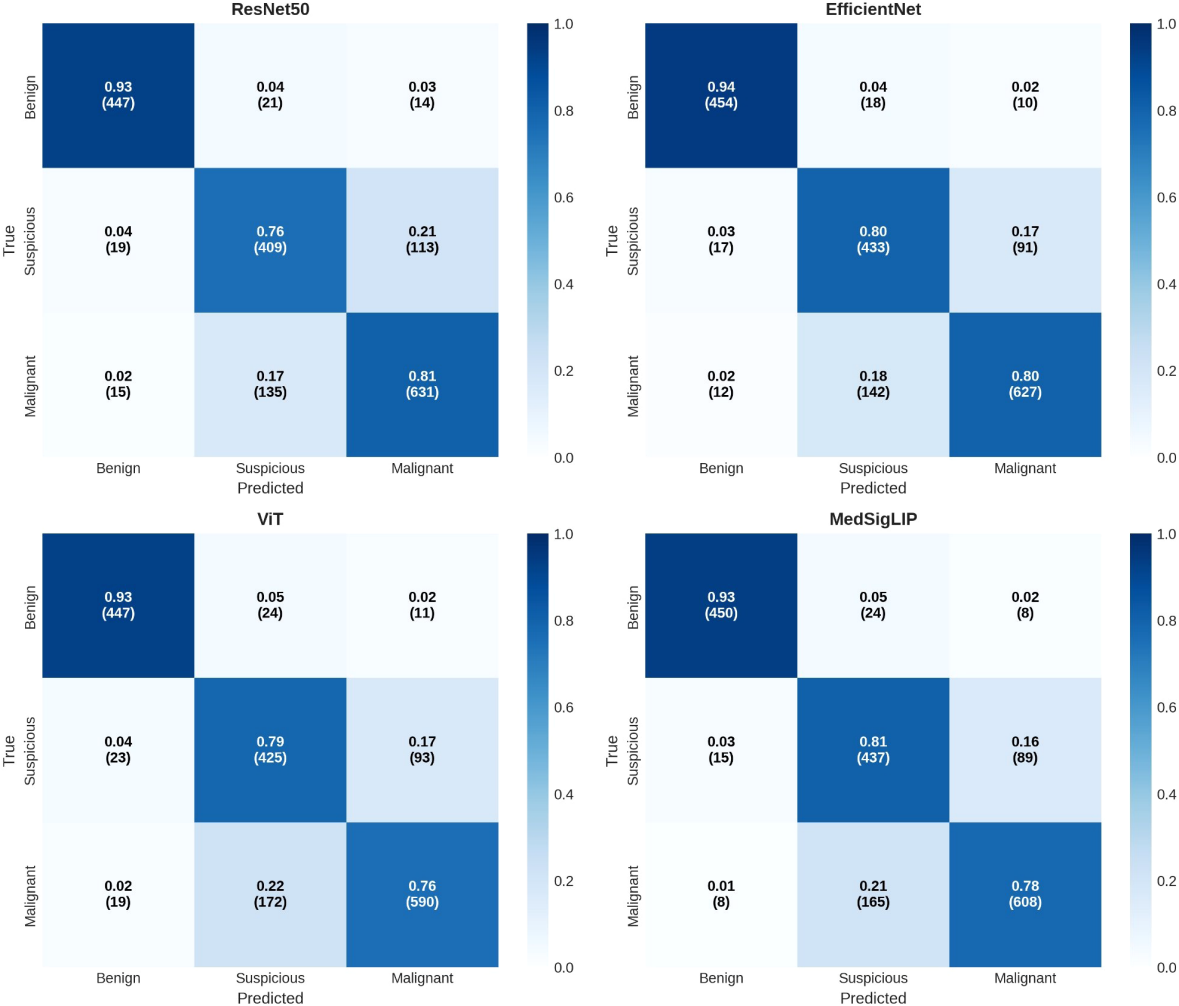
Out-of-fold confusion matrices across frozen encoders. Confusion matrices were computed from out-of-fold (OOF) predictions aggregated across the five cross-validation folds for ResNet50, EfficientNet-B0, ViT-Base, and MedSigLIP. Rows indicate the true class (Bethesda II/V/VI; Benign/Suspicious/Malignant) and columns indicate the predicted class. Each cell reports the proportion of samples and the corresponding count (in parentheses). Across encoders, the dominant error pattern reflects confusion between Bethesda V (Suspicious) and Bethesda VI (Malignant), whereas Bethesda II (Benign) is comparatively stable. OOF, out-of-fold.

Confusion matrices for all encoders are presented in **Figure 3**. Across models, errors were concentrated in the boundary between Suspicious (Bethesda V) and Malignant (Bethesda VI), whereas Benign predictions were comparatively stable.

Benign cases were correctly identified in the majority of instances across all encoders (447–454 of 482), with relatively few Benign to Malignant errors (8–14 of 482). The most common misclassification pattern involved Suspicious cases being predicted as Malignant (89–113 of 541) and Malignant cases being predicted as Suspicious (135–172 of 781), reflecting the known morphological continuum between borderline and malignant cytology.

### 3.4 Bethesda V (suspicious) detailed analysis

Bethesda V remained the most challenging class across encoders (Suspicious F1: 0.732–0.764; **Table 3**). Detailed analysis of Bethesda V performance is provided in **Table 4**. MedSigLIP achieved the highest recall for Suspicious cases (0.808), followed by EfficientNet (0.800), ViT (0.786), and ResNet50 (0.756).

**Table 4:** Focused analysis of the Suspicious class (Bethesda V) based on out-of-fold (OOF) predictions.

Encoder	Suspicious recall	S→benign rate	S→malignant rate	Mean P(Suspicious)
ResNet50	0.756	0.035 (19/541)	0.209 (113/541)	0.69
EfficientNet	0.8	0.031 (17/541)	0.168 (91/541)	0.743
ViT	0.786	0.043 (23/541)	0.172 (93/541)	0.735
MedSigLIP	0.808	0.028 (15/541)	0.165 (89/541)	0.723

S→Benign and S→Malignant denote the proportion (and count) of true Suspicious (Bethesda V; n=541) cases misclassified as Benign (Bethesda II) or Malignant (Bethesda VI), respectively. Mean P(Suspicious) is the mean predicted probability assigned to the Suspicious class among true Suspicious cases (n=541). S, Suspicious; P, predicted probability.

Misclassification rates revealed that a nontrivial fraction of Suspicious cases were predicted as Malignant, ranging from 16.5% to 20.9% across encoders (S→Malignant: 89/541 to 113/541; **Table 4**). In contrast, Suspicious to Benign misclassification was uncommon (2.8%–4.3%; 15/541 to 23/541).

**Figure 4:**
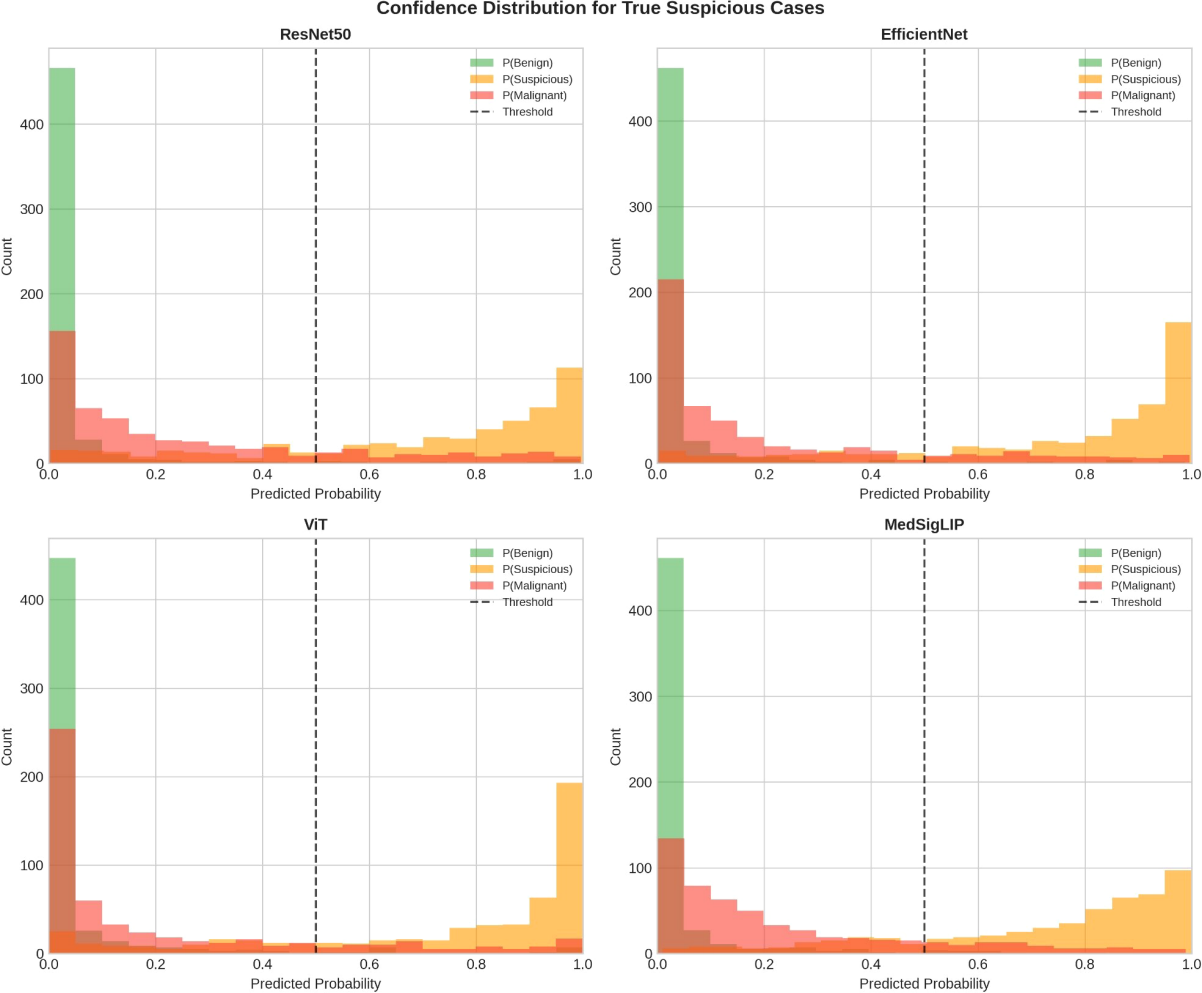
Confidence distributions for true Suspicious (Bethesda V) cases across encoders. Overlaid histograms show the predicted class probabilities assigned to Benign (P(Benign)), Suspicious (P(Suspicious)), and Malignant (P(Malignant)) among true Suspicious (Bethesda V) cases, stratified by encoder (ResNet50, EfficientNet-B0, ViT-Base, and MedSigLIP). The vertical dashed line marks the decision threshold (0.5). Differences in these distributions illustrate how each encoder allocates confidence and ambiguity for the borderline Bethesda V category, complementing class-wise performance (**Figure 2**) and error patterns (**Figure 3**; **Table 4**). P(·), predicted probability.

Model confidence behavior for the Suspicious class is illustrated in **Figure 4**. Mean predicted probability for the Suspicious class was highest for EfficientNet (mean P(Suspicious) = 0.743), followed by ViT (0.735), MedSigLIP (0.723), and ResNet50 (0.690) (**Table 4**), indicating systematic differences in how models distribute uncertainty around this borderline category. Mean predictive entropy for true Suspicious cases was highest for MedSigLIP (0.470 ± 0.241) and lowest for ViT (0.364 ± 0.276), with EfficientNet (0.387 ± 0.255) and ResNet50 (0.445 ± 0.244) showing intermediate values. Higher entropy in MedSigLIP reflects more distributed probability mass across classes for borderline cases, consistent with its conservative classification behavior and highest Suspicious recall (0.808).

### 3.5 Robustness and ablation studies

Training dynamics for a representative fold are shown in **Supplementary Figure 1**. Across encoders, validation macro-F1 typically plateaued after early epochs while training loss continued to decrease, consistent with mild overfitting controlled by early stopping.

Robustness testing under common image perturbations is summarized in **Supplementary Figure 2** and Supplementary Results. In a representative single-split evaluation (**Supplementary Table 2**), performance remained relatively stable under moderate perturbations (e.g., brightness increase and JPEG compression), while stronger degradations such as brightness decrease produced a noticeable reduction in macro-F1 (from 0.859 to 0.808).

Ablation results (**Supplementary Table 1**) confirmed that the MLP head substantially outperformed a linear classifier (macro-F1: 0.8416 ± 0.0160 vs. 0.8052 ± 0.0089). Removing class weights resulted in similar average macro-F1 but increased variability across folds.

Finally, calibration analysis demonstrated substantial differences in confidence reliability across encoders (**Supplementary Figure 3**). MedSigLIP exhibited the lowest Expected Calibration Error (ECE = 0.025), whereas ViT showed the highest miscalibration (ECE = 0.082). ResNet50 and EfficientNet displayed intermediate ECE values (0.044 and 0.052, respectively).

Adaptive ECE (ACE) results were consistent with fixed-bin ECE across all encoders (MedSigLIP: 0.0259; ViT: 0.0805), confirming that calibration differences were independent of binning strategy. Multiclass Brier Score corroborated these findings, with MedSigLIP achieving the lowest mean score (0.0782) and ViT the highest (0.0936). Class-wise calibration analysis for Bethesda V further confirmed MedSigLIP’s superior reliability in this diagnostically critical category (ECE = 0.0411, ACE = 0.0411; **Supplementary Table 3**; **Supplementary Figure 4**). Reliability diagrams (**Supplementary Figure 3**) reveal distinct calibration patterns across encoders. MedSigLIP’s curves closely track the perfect calibration diagonal across all three classes. EfficientNet and ResNet50 show moderate over-confidence at intermediate probability ranges. ViT exhibits the most pronounced deviation, with substantial over-confidence particularly in mid-range bins. Class-wise reliability diagrams for Bethesda V (**Supplementary Figure 4**) further show that MedSigLIP maintains near-diagonal calibration for the suspicious class, while ViT displays irregular confidence patterns that deviate substantially from perfect calibration.

## 4 Discussion

### 4.1 Medical pretraining does not guarantee superior accuracy: reconsidering domain specificity

This study benchmarked four frozen visual encoders on three-class thyroid FNAB cytology classification (Bethesda II/V/VI). EfficientNet achieved the highest macro-F1 (0.845 ± 0.021), followed closely by MedSigLIP (0.836 ± 0.019), ResNet50 (0.829 ± 0.015), and ViT (0.817 ± 0.020) (**Table 3**; **Figure 2**). Importantly, paired testing showed that EfficientNet significantly outperformed ViT, whereas the difference between EfficientNet and MedSigLIP was not statistically significant after multiple comparison correction (**Table 1**). Therefore, the observed accuracy gap between EfficientNet and MedSigLIP should be interpreted as a numerical difference rather than definitive superiority. The absolute macro-F1 difference between the two models was approximately 0.8% (Δ = 0.0084; 95% CI [−0.009, +0.026]), a magnitude unlikely to be clinically meaningful in practice. Accordingly, encoder choice for thyroid cytology workflow support should be framed as a trade-off between discrimination and reliability, rather than a single “best model” claim.

Several factors may explain why medical pretraining did not translate into clear accuracy gains in this cytology setting. Deep learning approaches for thyroid disease classification have shown promising results across various imaging modalities, including ultrasound-based hybrid architectures (35). First, “medical” pretraining corpora may be dominated by radiology like distributions and may not fully capture the microscopy specific cues central to cytopathology (e.g., nuclear chromatin texture, nuclear to cytoplasmic ratio, and subtle architectural arrangements). Second, prior work suggests that strong ImageNet representations can transfer effectively to medical tasks and that performance gains may depend more on optimization and representation robustness than on strict domain matching (15, 18, 22). Third, in a frozen-encoder benchmark, representational quality is tested under a constrained adaptation regime; it is possible that full fine-tuning or domain adaptive training would yield different relative rankings. Taken together, our results argue for empirical encoder selection in cytology rather than assuming domain-specific pretraining will always be superior.

### 4.2 The persistent challenge of Bethesda V and the nature of borderline cytology

Across all encoders, Bethesda V (Suspicious) was the most difficult category (F1: 0.732–0.764) compared to benign and malignant classes (**Table 3**). Error analysis showed that a sizable portion of Suspicious cases were predicted as Malignant (16.5–20.9%), whereas Suspicious to Benign errors were less frequent (2.8–4.3%) (**Table 4**). This pattern is clinically plausible: Bethesda V represents a morphologic continuum where aspirates can exhibit partial malignant features without meeting definitive criteria (5, 6). Consequently, even high performing models may struggle to sharply separate Suspicious from Malignant using image only features, reflecting the intrinsic ambiguity of the category rather than purely algorithmic failure. From a clinical risk perspective, Suspicious-to-Malignant misclassification (16.5–20.9%) may represent ‘acceptable conservatism’ rather than pure algorithmic failure, as it reflects the inherent morphological continuum between Bethesda V and VI and the known malignancy risk of Bethesda V (estimated at 60–75% in contemporary series (5)). In contrast, Suspicious-to-Benign misclassification (2.8–4.3%) carries a higher clinical risk of missed malignancy. The asymmetry of these error costs — where false reassurance is more dangerous than false alarm — supports the prioritization of Suspicious recall over precision in AI-assisted triage workflows.

Notably, MedSigLIP achieved the highest recall for Suspicious cases (0.808), closely followed by EfficientNet (0.800) (**Table 4**). In practical terms, high recall in Bethesda V may be attractive for clinical decision support when the goal is to avoid missing suspicious lesions; however, this must be balanced against the downstream consequences of shifting errors toward malignant predictions. Our confusion matrices (**Figure 3**) and detailed suspicious analysis (**Table 4**) suggest that the borderline region remains the dominant error source and is the most relevant target for future methodological improvement (e.g., multimodal integration or uncertainty aware triage workflows).

### 4.3 Calibration as a critical deployment criterion

Beyond accuracy, reliability of model confidence is essential for safe deployment. We observed substantial calibration differences across encoders: MedSigLIP produced the lowest Expected Calibration Error (ECE = 0.025), whereas ViT exhibited the highest miscalibration (ECE = 0.082), with ResNet50 and EfficientNet showing intermediate values (ECE = 0.044 and 0.052, respectively) (**Supplementary Figure 3**). While no universal threshold exists, ECE values below 0.05 have been used as a practical benchmark for well-calibrated models in medical imaging applications (24), and MedSigLIP’s ECE of 0.025 compares favorably with values reported in comparable studies.This finding suggests that medical pretraining may provide an advantage in probability reliability even when classification accuracy is similar.

Prior studies have shown that modern neural networks—particularly Transformers—may be overconfident and poorly calibrated, which can be hazardous in clinical decisionmaking (24). Uncertainty aware approaches have been proposed to mitigate these risks and improve decision support in medical imaging (25, 26). In our setting, calibration quality is particularly relevant for Bethesda V, where clinical decisions depend heavily on recognizing borderline morphology. A well-calibrated model like MedSigLIP can support threshold-based triage (e.g., flagging low confidence cases for mandatory expert review) rather than presenting confidence as a misleading proxy for correctness.

### 4.4 Robustness, training dynamics, and practical considerations

Training curves from a representative fold (**Supplementary Figure 1**) show rapid early improvement in validation macro-F1 followed by plateauing while training loss continues to decline, consistent with mild overfitting controlled by early stopping. This supports the adopted training protocol for the lightweight head on frozen embeddings.

In robustness testing (**Supplementary Figure 2**) and a representative single-split analysis (**Supplementary Table 2**), performance remained relatively stable under moderate perturbations, while more severe degradations—specifically a 20% decrease in brightness—produced a clear performance decline (macro-F1 dropped to 0.808). These results underscore a key practical consideration for cytology deployment: variability in slide preparation, staining intensity, illumination, and imaging pipelines can introduce domain shifts. Therefore, encoder choice should consider not only average accuracy but also calibration and robustness under realistic acquisition variability.

Ablation analysis (**Supplementary Table 1**) further supports the benchmark design: the MLP head outperformed a linear classifier (macro-F1: 0.842 ± 0.016 vs. 0.805 ± 0.009), indicating that a modest nonlinear head better exploits frozen representations. Removing class weights yielded similar mean macro-F1 but increased variability across folds, suggesting that fold-wise class weighting contributes to stability under imbalance (**Supplementary Table 1**).

### 4.5 Limitations

This study has several limitations. First, we evaluated a three-class formulation (Bethesda II, V, VI), which excludes other clinically relevant categories (Bethesda I, III, IV) and therefore does not represent a complete real-world thyroid cytology triage system (6–8). From a clinical triage perspective, the Bethesda II/V/VI formulation corresponds to the most actionable decision points: confident benign (surveillance), suspicious (surgery or close follow-up), and malignant (definitive surgery). Bethesda III and IV represent indeterminate categories where molecular testing is typically employed before AI-assisted classification would be meaningful. Second, although each image corresponds to a unique patient case (mitigating patient level leakage), the dataset still represents a specific acquisition setting and may not capture broader inter-institutional heterogeneity in staining, slide preparation, and imaging devices. Specifically, all images in ThyroidEffi 1.0 were acquired using a single staining protocol (Diff-Quik), a single microscope model (Olympus BX43), and a single magnification (40×). In real-world deployment, staining batch variability, scanner heterogeneity, and inter-institutional differences in slide preparation protocols would introduce domain shifts not captured in this benchmark.

Third, the benchmark used frozen encoders; thus, results reflect representation transfer under constrained adaptation and may differ under full fine-tuning or domain adaptive training strategies. Full fine-tuning was intentionally excluded to isolate representational quality; it is possible that end-to-end optimization would alter the observed ranking, particularly for domain-specific encoders such as MedSigLIP. Similarly, the resolution difference between MedSigLIP (448×448) and other encoders (224×224) follows native preprocessing protocols but represents a mild benchmarking confound that cannot be fully eliminated in a frozen-encoder setting. Fourth, we did not incorporate multimodal clinical variables (e.g., ultrasound features, molecular markers, or clinical context) that often inform real FNAB decision making and could be especially valuable for resolving borderline categories (7, 8). Fifth, we did not include explainability analysis (e.g., saliency or attention maps), which may be important for clinician trust and for identifying spurious model cues. Explainability methods such as Grad-CAM and attention maps were considered but excluded from the current benchmark scope; Grad-CAM is not directly applicable to frozen encoder embeddings used as MLP inputs, as attribution maps would reflect the MLP head rather than the encoder features. Their rigorous implementation in a frozen-encoder setting requires additional methodological steps and remains entirely future work.Finally, external validation on independent datasets is necessary before clinical translation, particularly for Bethesda V where ambiguity is intrinsic and calibration properties are critical.

### 4.6 Future directions

These findings motivate several directions for future work. Multi center external validation across diverse staining protocols, microscopes, and acquisition pipelines is essential to establish generalizability. Direct comparison of frozen versus fine-tuned strategies could clarify whether domain-specific encoders such as MedSigLIP yield larger gains when allowed to adapt to cytology specific textures and morphologies. Incorporating multimodal information (cytology images plus ultrasound, molecular data, and clinical context) may be particularly promising for improving performance and interpretability in borderline categories.

In parallel, uncertainty aware modeling should be prioritized. Approaches such as Bayesian methods, deep ensembles, or evidential learning may better identify diagnostically ambiguous cases and enable safer human–AI collaboration, particularly for Bethesda V where confident misclassification is a key risk (25, 26). Finally, prospective workflow studies—evaluating AI-assisted triage and its impact on diagnostic accuracy, turnaround time, and interobserver variability—will be necessary to demonstrate clinical utility. The frozen encoder embeddings generated in this benchmark are naturally amenable to multimodal integration. Since embeddings are fixed-dimensional vector representations (e.g., 1152-d for MedSigLIP), they can be concatenated or fused with embeddings from other modalities — such as ultrasound image features, molecular marker vectors, or structured clinical variables — within a joint classification head. This modularity makes the current pipeline a potential building block for future multimodal thyroid triage systems.

## 5 Conclusions

In this frozen-encoder benchmark on three-class thyroid FNAB cytology (Bethesda II/V/VI; N = 1,804), EfficientNet achieved the highest macro-F1 (0.845 ± 0.021), followed closely by MedSigLIP (0.836 ± 0.019), ResNet50 (0.829 ± 0.015), and ViT (0.817 ± 0.020) (**Table 3**; **Figure 2**). Pairwise testing indicated that EfficientNet significantly outperformed ViT, while the difference between EfficientNet and MedSigLIP was not statistically significant after multiple comparison correction (**Table 1**).

Across all encoders, Bethesda V (Suspicious) remained the most challenging class (F1: 0.732–0.764), with a consistent tendency for suspicious cases to be predicted as malignant (16.5–20.9%) (**Table 4**; **Figure 3**). MedSigLIP showed strong sensitivity for Bethesda V (recall = 0.808) and the best calibration (ECE = 0.025), highlighting that calibration and uncertainty behavior can meaningfully differ even when accuracy is similar (**Supplementary Figure 3**).

Overall, our findings suggest that domain specific medical pretraining does not guarantee superior classification accuracy for thyroid cytology in a frozen-encoder setting, and that encoder selection for clinical decision support should consider calibration, robustness, and borderline category behavior alongside aggregate performance metrics. In particular, well-calibrated models such as MedSigLIP suggest a potential benefit in reducing overconfident misclassification in borderline Bethesda V cases, pending prospective validation in real-world triage workflows.

## Data Availability

The datasets presented in this study can be found in online repositories. The names of the repository/repositories and accession number(s) can be found below: The code used for this benchmark (data preprocessing, model definitions, and evaluation scripts) is available at GitHub (https://github.com/opisthion06/ENCODERS) and archived on Zenodo (doi:10.5281/zenodo.18378561). The ThyroidEffi 1.0 dataset analyzed in this study is available upon reasonable request from the corresponding author of the original publication (30).

## References

[1] Haugen BR Alexander EK Bible KC Doherty GM Mandel SJ Nikiforov YE . 2015 American Thyroid Association management guidelines for adult patients with thyroid nodules and differentiated thyroid cancer. Thyroid. (2016) 26:1–133. doi: 10.1089/thy.2015.0020. PMID: 26462967 PMC4739132

[2] Koelliker EL Krumeich LN Kravchenko T Keamy Blanco MM Letica-Kriegel AS Hsu I . Sonographic and pathologic features of Malignant hot thyroid nodules: a multi-institutional study. Surgery. (2026) 189:109710. doi: 10.1016/j.surg.2025.109710. PMID: 41062395

[3] Valderrabano P Khazai L Thompson ZJ Otto KJ Hallanger-Johnson JE Wadsworth JT . Cancer risk stratification of indeterminate thyroid nodules: a cytological approach. Thyroid. (2017) 27:1277–84. doi: 10.1089/thy.2017.0221. PMID: 28806881 PMC6112164

[4] Lim H Devesa SS Sosa JA Check D Kitahara CM . Trends in thyroid cancer incidence and mortality in the United States, 1974–2013. JAMA. (2017) 317:1338–48. doi: 10.1001/jama.2017.2719. PMID: 28362912 PMC8216772

[5] Cibas ES Ali SZ . The 2017 bethesda system for reporting thyroid cytopathology. Thyroid. (2017) 27:1341–6. doi: 10.1089/thy.2017.0500. PMID: 29091573

[6] Bongiovanni M Spitale A Faquin WC Mazzucchelli L Baloch ZW . The bethesda system for reporting thyroid cytopathology: a meta-analysis. Acta Cytol. (2012) 56:333–9. doi: 10.1159/000339959. PMID: 22846422

[7] Nicholson KJ Roberts MS McCoy KL Carty SE Yip L . Molecular testing versus diagnostic lobectomy in Bethesda III/IV thyroid nodules: a cost-effectiveness analysis. Thyroid. (2019) 29:1237–43. doi: 10.1089/thy.2018.0779. PMID: 31407625 PMC7366255

[8] Nacchio M Palladino R Vigliar E Pisapia P Salatiello M Malapelle U . Evaluating local thyroid cytopathology practices by molecular quality metrics: a multi-institutional study on 4651 FNAs with a focus on the role of the interventional cytopathologist. Cancer Cytopathol. (2023) 131:772–80. doi: 10.1002/cncy.22756. PMID: 37635646

[9] Bang H Cho C Kim MY Hyeon J Lee SE . Subclassifying “Atypia of Undetermined Significance (AUS)” category in the 2023 Bethesda System for Thyroid Cytopathology: analyzing K-TIRADS, BRAF V600E mutation, and risk of Malignancy. Endocr Pathol. (2025) 36:12. doi: 10.1007/s12022-025-09856-1. PMID: 40232600

[10] Negrelli M Frascarelli C Maffini F Mangione E Di Tonno C Lombardi M . Artificial intelligence in thyroid cytopathology: diagnostic and technical insights. Cancers. (2025) 17:3525. doi: 10.3390/cancers17213525. PMID: 41228318 PMC12610226

[11] Park VY Han K Seong YK Park MH Kim EK Moon HJ . Diagnosis of thyroid nodules: performance of a deep learning convolutional neural network model vs. radiologists. Sci Rep. (2019) 9:17843. doi: 10.1038/s41598-019-54434-1. PMID: 31780753 PMC6882804

[12] Kim J Kim M-H Lim D-J Lee H Lee JJ Kwon H-S . Deep learning technology for classification of thyroid nodules using multi-view ultrasound images: potential benefits and challenges in clinical application. Endocrinol Metab (Seoul). (2025) 40:216–24. doi: 10.3803/EnM.2024.2058. PMID: 39805576 PMC12061742

[13] Sanyal P Mukherjee T Barui S Das A Gangopadhyay P . Artificial intelligence in cytopathology: a neural network to identify papillary carcinoma on thyroid fine-needle aspiration cytology smears. J Pathol Inform. (2018) 9:43. doi: 10.4103/jpi.jpi_43_18. PMID: 30607310 PMC6289006

[14] Range DD Dov D Kovalsky SZ Henao R Carin L Cohen J . Application of a machine learning algorithm to predict Malignancy in thyroid cytopathology. Cancer Cytopathol. (2020) 128:287–95. doi: 10.1002/cncy.22238. PMID: 32012493

[15] Raghu M Zhang C Kleinberg J Bengio S . (2019). Transfusion: understanding transfer learning for medical imaging, in: Advances in Neural Information Processing Systems, (San Diego, CA: Neural Information Processing Systems Foundation, Inc.).

[16] Deng J Dong W Socher R Li L-J Li K Fei-Fei L . (2009). ImageNet: a large-scale hierarchical image database, in: Proceedings of the IEEE Conference on Computer Vision and Pattern Recognition (CVPR), (Piscataway, NJ: IEEE) pp. 248–55. doi: 10.1109/CVPR.2009.5206848, PMID:

[17] Ridnik T Ben-Baruch E Noy A Zelnik-Manor L . (2021). ImageNet-21K pretraining for the masses, in: Advances in Neural Information Processing Systems (NeurIPS) 2021, Datasets and Benchmarks Track, (San Diego, CA: Neural Information Processing Systems Foundation, Inc.).

[18] Kornblith S Shlens J Le QV . (2019). Do better ImageNet models transfer better?, in: Proceedings of the IEEE/CVF Conference on Computer Vision and Pattern Recognition (CVPR), (Piscataway, NJ: IEEE). pp. 2661–71. doi: 10.1109/CVPR.2019.00277, PMID:

[19] Google for Developers . MedSigLIP model card. Health AI developer foundations. Available online at: developers.google.com/health-ai-developer-foundations/medsiglip/model-card (Accessed January 10, 2026).

[20] Sellergren A Kazemzadeh S Jaroensri T Kiraly A Traverse M Kohlberger T . MedGemma technical report. arXiv. (2025) arXiv:2507.05201. doi: 10.48550/arXiv.2507.05201. PMID: 41363103

[21] Zhai X Mustafa B Kolesnikov A Beyer L . (2023). Sigmoid loss for language image pre-training, in: Proceedings of the IEEE/CVF International Conference on Computer Vision (ICCV), (Piscataway, NJ: IEEE). pp. 11975–86. doi: 10.1109/ICCV51070.2023.01100, PMID:

[22] Azizi S Mustafa B Ryan F Beaver Z Freyberg J Deaton J . (2021). Big self-supervised models advance medical image classification, in: Proceedings of the IEEE/CVF International Conference on Computer Vision (ICCV), (Piscataway, NJ: IEEE). doi: 10.1109/ICCV48922.2021.00346, PMID:

[23] Hita X Javed F Lodi S . Reliable leukemia detection via transfer-enhanced Bayesian CNNs. Comput Biol Med. (2026) 202:111419. doi: 10.1016/j.compbiomed.2025.111419. PMID: 41485393

[24] Guo C Pleiss G Sun Y Weinberger KQ . (2017). On calibration of modern neural networks, in: Proceedings of the 34th International Conference on Machine Learning (ICML) PMLR, (Cambridge, MA: JMLR), Vol. 70. pp. 1321–30.

[25] Casali N Brusaferri A Baselli G Fumagalli S Micotti E Forloni G . A comprehensive framework for uncertainty quantification of voxel-wise supervised deep learning models in IVIM MRI. NMR BioMed. (2026) 39:e70227. doi: 10.1002/nbm.70227. PMID: 41555683

[26] Damiano R Lanzarone E Lussana F Villa G Daniel AJ Francis S . The impact of uncertainty estimation on radiomic segmentation reproducibility and scan-rescan repeatability in kidney MRI. Med Phys. (2025) 52:e17995. doi: 10.1002/mp.17995. PMID: 40665574 PMC12264328

[27] He K Zhang X Ren S Sun J . (2016). Deep residual learning for image recognition, in: Proceedings of the IEEE Conference on Computer Vision and Pattern Recognition (CVPR), (Piscataway, NJ: IEEE). pp. 770–8. doi: 10.1109/CVPR.2016.90, PMID:

[28] Tan M Le QV . (2019). EfficientNet: rethinking model scaling for convolutional neural networks, in: Proceedings of the 36th International Conference on Machine Learning (ICML) PMLR, (Cambridge, MA: JMLR), Vol. 97. pp. 6105–14.

[29] Dosovitskiy A Beyer L Kolesnikov A Weissenborn D Zhai X Unterthiner T . (2021). An image is worth 16×16 words: transformers for image recognition at scale, in: Proceedings of the International Conference on Learning Representations (ICLR), ( OpenReview.net).

[30] Pham-Ngoc H Nguyen-Van D Vu-Tien D Le-Hong P . ThyroidEffi 1.0: a cost-effective system for high-performance multi-class thyroid carcinoma classification. arXiv. (2025) arXiv:2504.14139. doi: 10.48550/arXiv.2504.14139. PMID: 41363103

[31] Efron B Tibshirani RJ . An introduction to the bootstrap. New York: Chapman & Hall/CRC (1993). doi: 10.1201/9780429246593

[32] McNemar Q . Note on the sampling error of the difference between correlated proportions or percentages. Psychometrika. (1947) 12:153–7. doi: 10.1007/BF02295996. PMID: 20254758

[33] Benjamini Y Hochberg Y . Controlling the false discovery rate: a practical and powerful approach to multiple testing. J R Stat Soc Ser B (Methodol). (1995) 57:289–300. doi: 10.1111/j.2517-6161.1995.tb02031.x. PMID: 41858021

[34] Hendrycks D Dietterich T . (2019). Benchmarking neural network robustness to common corruptions and perturbations, in: Proceedings of the International Conference on Learning Representations (ICLR), ( OpenReview.net).

[35] Haider ZA Alsadhan NA Khan FM Al-Azzawi W Khan IU Ullah I . Deep learning-based dual optimization framework for accurate thyroid disease diagnosis using CNN architectures. Mehran Univ Res J Eng Technol. (2025) 44:1–12. doi: 10.22581/muet1982.0035

