# Supplementary material for: An early evaluation of MedSigLIP in thyroid cytology: a comparative frozen-encoder benchmark against ImageNet-pretrained encoders
Source: Front Endocrinol (Lausanne). 2026 Apr 10;17:1800630. doi: 10.3389/fendo.2026.1800630 (PMC13106084; doi:10.3389/fendo.2026.1800630)
Supplement: Supplementary file 1 [file DataSheet1.docx]

***SUPPLEMENTARY MATERIAL***

**1. Supplementary Figures and Tables**

**1.1 Supplementary Tables**

**Table S1.** Ablation analysis of classifier-head configuration (EfficientNet).

| **Configuration** | **Macro-F1** |
| --- | --- |
| MLP Head + Class Weights (Baseline) | 0.8416 ± 0.0160 |
| Linear Head + Class Weights | 0.8052 ± 0.0089 |
| MLP Head + No Class Weights | 0.8439 ± 0.0254 |

Values are mean ± SD across folds where applicable. Baseline corresponds to the default head and weighting used in the main benchmark.

**Table S2.** Single stratified train/test split sensitivity analysis.

| **Encoder** | **Macro-F1** | **Balanced Acc** | **Benign F1** | **Suspicious F1** | **Malignant F1** |
| --- | --- | --- | --- | --- | --- |
| ResNet50 | 0.8204 | 0.8237 | 0.953 | 0.6994 | 0.8087 |
| EfficientNet | 0.8578 | 0.8576 | 0.9583 | 0.7665 | 0.8485 |
| ViT | 0.8513 | 0.8493 | 0.9379 | 0.7673 | 0.8487 |
| MedSigLIP | 0.839 | 0.8464 | 0.9333 | 0.7602 | 0.8235 |

Metrics computed on a single held-out test split. This analysis complements cross-validation results and assesses ranking stability.

**Table S3.** Extended calibration metrics across encoders.

| **Encoder** | **ECE (fixed)** | **ACE (adaptive)** | **Brier Score** | **ECE-Susp** | **ACE-Susp** |
| --- | --- | --- | --- | --- | --- |
| ResNet50 | 0.0444 | 0.0427 | 0.0855 | 0.0437 | 0.0402 |
| EfficientNet | 0.0519 | 0.0527 | 0.0805 | 0.0505 | 0.0478 |
| ViT | 0.0819 | 0.0805 | 0.0936 | 0.0779 | 0.0683 |
| MedSigLIP | 0.0254 | 0.0259 | 0.0782 | 0.0411 | 0.0411 |

ECE: fixed-bin Expected Calibration Error; ACE: Adaptive Calibration Error (equal-mass bins); Brier: mean multiclass Brier Score; ECE-Susp/ACE-Susp: class-wise calibration for Bethesda V.


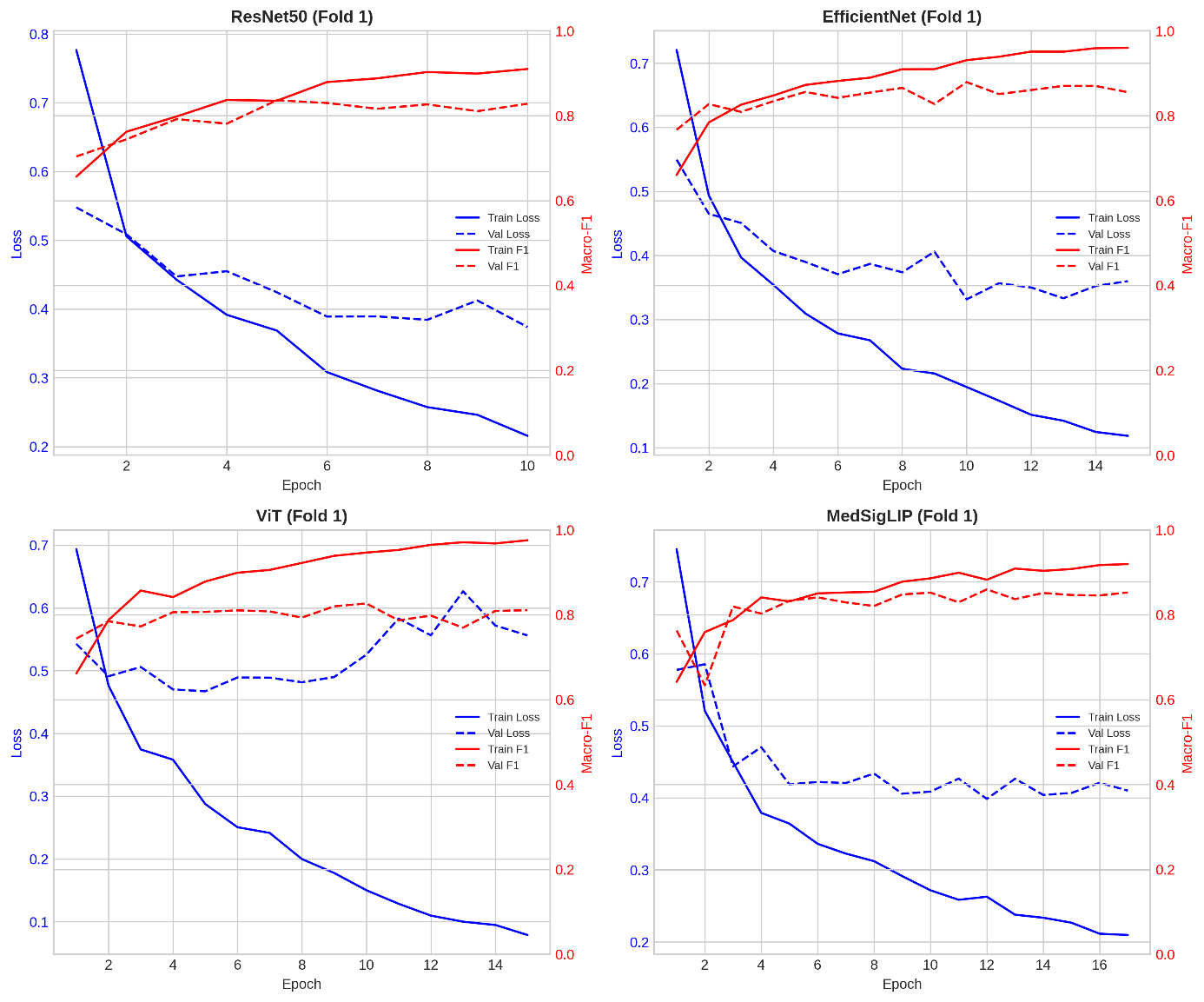
**1.2 Supplementary Figures**

**Figure S1. Training dynamics of the classifier head (representative fold).** Training curves from a representative cross-validation fold illustrate the optimization behavior of the MLP classifier head trained on frozen encoder embeddings. The plot shows training loss and validation macro-F1 across epochs, with early stopping based on validation performance. Validation macro-F1 typically plateaus earlier than training loss, indicating limited overfitting that is mitigated by early stopping. Abbreviations: MLP, multilayer perceptron.


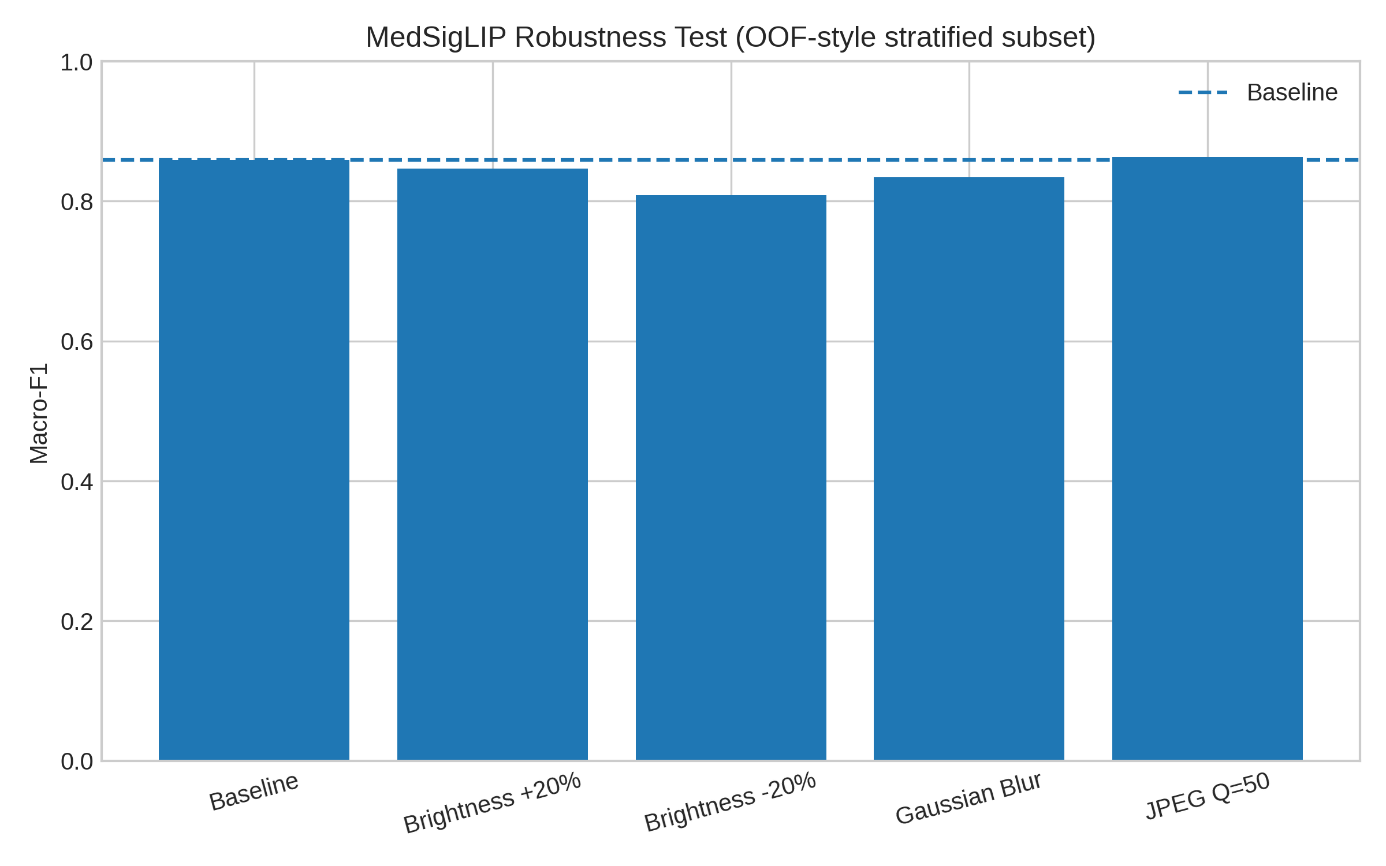
**Figure S2. Robustness under common image perturbations.** Robustness to common image perturbations (e.g., brightness shifts, Gaussian blur, and JPEG compression) was evaluated by measuring changes in macro-F1 relative to the unperturbed condition. Results are reported for each encoder to illustrate sensitivity to acquisition- and compression-related variability. Detailed numeric results from the representative single stratified train/test split setting are provided in Table S2. Abbreviations: OOF, out-of-fold.


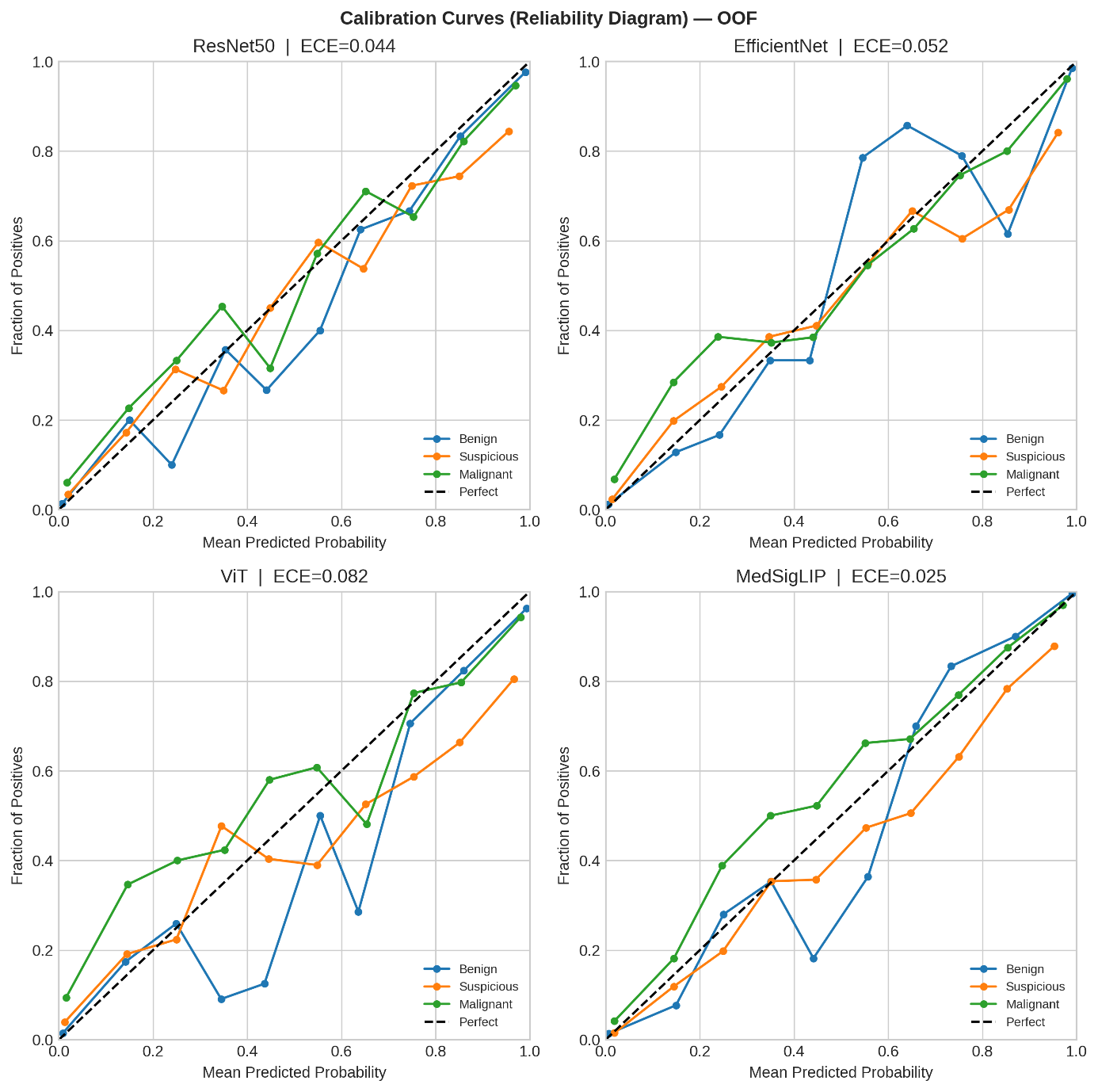
**Figure S3. Calibration analysis and reliability diagrams**. Calibration was assessed for each encoder using expected calibration error (ECE) and reliability diagrams. Reliability diagrams plot empirical accuracy against predicted confidence across confidence bins, allowing visualization of agreement between confidence and correctness. Lower ECE indicates better calibration. Numeric ECE values are summarized in the supplementary calibration results. Abbreviations: ECE, expected calibration error.

**
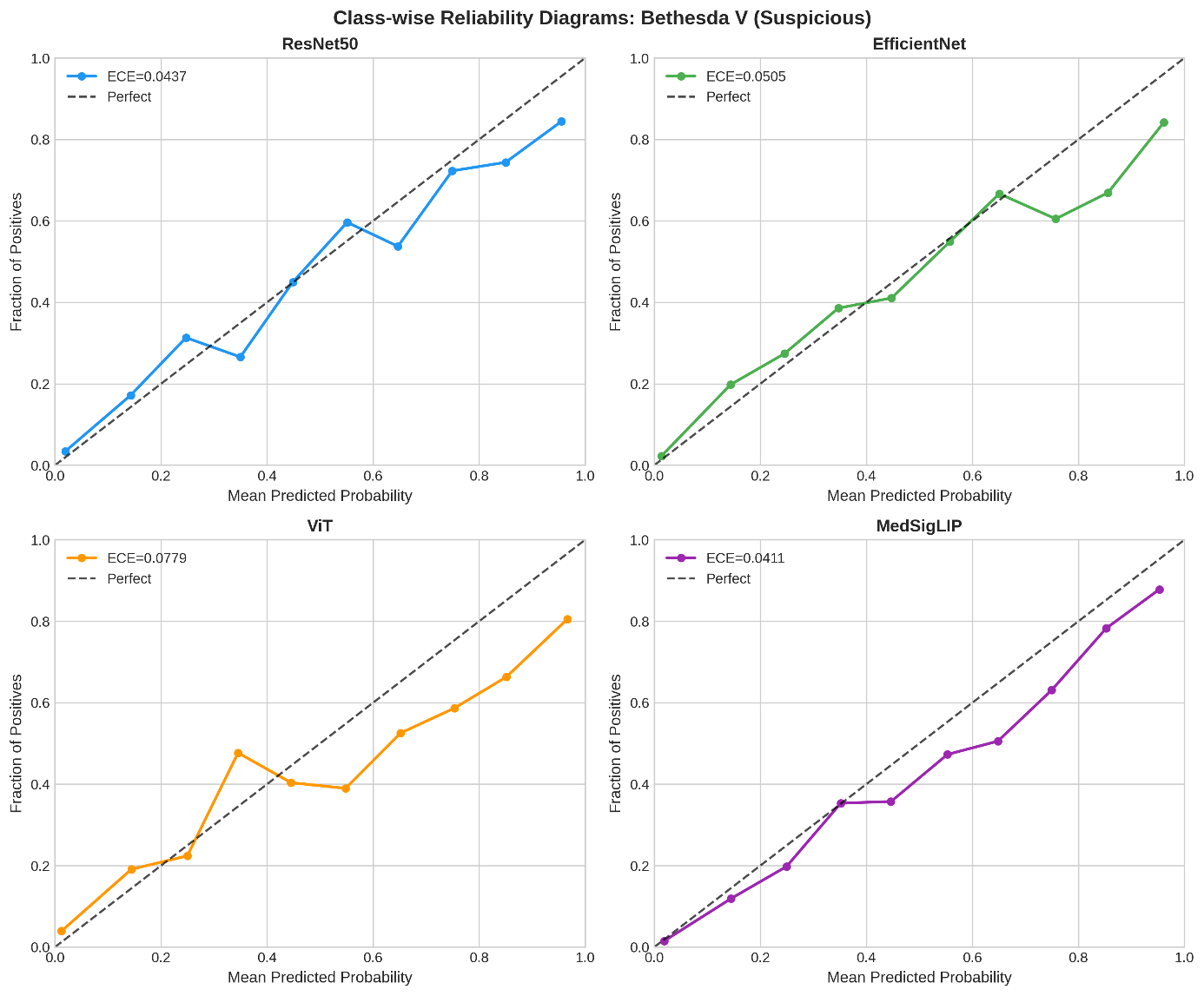
**

**Figure S4.** Class-wise reliability diagrams for Bethesda V (Suspicious). Calibration curves show empirical fraction of positives against mean predicted probability for the Suspicious class across encoders. MedSigLIP demonstrates the best-calibrated confidence estimates for this diagnostically critical category.
